# Effectiveness of Trivalent Inactivated Influenza Vaccine in Children Estimated by a Test-Negative Case-Control Design Study Based on Influenza Rapid Diagnostic Test Results

**DOI:** 10.1371/journal.pone.0136539

**Published:** 2015-08-28

**Authors:** Masayoshi Shinjoh, Norio Sugaya, Yoshio Yamaguchi, Yuka Tomidokoro, Shinichiro Sekiguchi, Keiko Mitamura, Motoko Fujino, Hiroyuki Shiro, Osamu Komiyama, Nobuhiko Taguchi, Yuji Nakata, Naoko Yoshida, Atsushi Narabayashi, Michiko Myokai, Masanori Sato, Munehiro Furuichi, Hiroaki Baba, Hisayo Fujita, Akihiro Sato, Ichiro Ookawara, Kenichiro Tsunematsu, Makoto Yoshida, Mio Kono, Fumie Tanaka, Chiharu Kawakami, Takahisa Kimiya, Takao Takahashi, Satoshi Iwata, Keio Pediatric Influenza Research Group

**Affiliations:** 1 Department of Pediatrics, Keio University School of Medicine, Shinjuku-ku, Tokyo, Japan; 2 Center for Infectious Diseases and Infection Control, Keio University School of Medicine, Shinjuku-ku, Tokyo, Japan; 3 Department of Paediatrics, Keiyu Hospital, Yokohama, Kanagawa, Japan; 4 Department of Clinical Research, National Hospital Organization, Utsunomiya, Tochigi Medical Center, Tochigi, Japan; 5 Department of Pediatrics, Tokyo Metropolitan Ohtsuka Hospital, Toshima-ku, Tokyo, Japan; 6 Department of Pediatrics, Eiju General Hospital, Taito-ku, Tokyo, Japan; 7 Department of Pediatrics, Saiseikai Central Hospital, Minato-ku, Tokyo, Japan; 8 Department of Pediatrics, Yokohama Rosai Hospital, Yokohama, Kanagawa, Japan; 9 Department of Pediatrics, National Hospital Organization, Tokyo Medical Center, Meguro-ku, Tokyo, Japan; 10 Department of Pediatrics, Nippon Kokan Hospital, Kawasaki, Kanagawa, Japan; 11 Department of Pediatrics, Kyosai Tachikawa Hospital, Tachikawa, Tokyo, Japan; 12 Department of Paediatrics, Kawasaki Municipal Hospital, Kawasaki, Kanagawa, Japan; 13 Department of Pediatrics, Shizuoka City Shimizu Hospital, Shizuoka, Shizuoka, Japan; 14 Department of Pediatrics, Tokyo Dental College Ichikawa General Hospital, Ichikawa, Chiba, Japan; 15 Department of Pediatrics, Saitama City Hospital, Saitama, Saitama, Japan; 16 Department of Pediatrics, Fuji Heavy Industries Health Insurance Society Ota Memorial Hospital, Ota, Gunma, Japan; 17 Department of Pediatrics, Hiratsuka Kyosai Hospital, Hiratsuka, Kanagawa, Japan; 18 Department of Pediatrics, Yokohama Municipal Citizen's hospital, Yokohama, Kanagawa, Japan; 19 Department of Pediatrics, Japanese Red Cross Shizuoka Hospital, Shizuoka, Shizuoka, Japan; 20 Department of Pediatrics, Hino Municipal Hospital, Hino, Tokyo, Japan; 21 Department of Pediatrics, Sano Kousei General Hospital, Sano, Tochigi, Japan; 22 Department of Pediatrics, National Hospital Organization Saitama National Hospital, Wako, Saitama, Japan; 23 Department of Pediatrics, Hiratsuka City Hospital, Hiratsuka, Kanagawa, Japan; 24 Yokohama City Institute of Health, Yokohama, Kanagawa, Japan; 25 Department of Infectious Diseases, Keio University School of Medicine, Shinjuku-ku, Tokyo, Japan; Melbourne School of Population Health, AUSTRALIA

## Abstract

We assessed vaccine effectiveness (VE) against medically attended, laboratory-confirmed influenza in children 6 months to 15 years of age in 22 hospitals in Japan during the 2013–14 season. Our study was conducted according to a test-negative case-control design based on influenza rapid diagnostic test (IRDT) results. Outpatients who came to our clinics with a fever of 38°C or over and had undergone an IRDT were enrolled in this study. Patients with positive IRDT results were recorded as cases, and patients with negative results were recorded as controls. Between November 2013 and March 2014, a total of 4727 pediatric patients (6 months to 15 years of age) were enrolled: 876 were positive for influenza A, 66 for A(H1N1)pdm09 and in the other 810 the subtype was unknown; 1405 were positive for influenza B; and 2445 were negative for influenza. Overall VE was 46% (95% confidence interval [CI], 39–52). Adjusted VE against influenza A, influenza A(H1N1)pdm09, and influenza B was 63% (95% CI, 56–69), 77% (95% CI, 59–87), and 26% (95% CI, 14–36), respectively. Influenza vaccine was not effective against either influenza A or influenza B in infants 6 to 11 months of age. Two doses of influenza vaccine provided better protection against influenza A infection than a single dose did. VE against hospitalization influenza A infection was 76%. Influenza vaccine was effective against influenza A, especially against influenza A(H1N1)pdm09, but was much less effective against influenza B.

## Introduction

Influenza vaccination is the most effective method of preventing influenza virus infection and its potentially severe complications, and vaccine efficacy from randomized control trials [1–3] and vaccine effectiveness (VE) from observational studies [4–7] in healthy children has been reported to be 40%-70%. However, VE varies considerably with time, place, and degree of antigenic distance between the vaccine strain and circulating strain. Although VE has generally been interpreted in the context of vaccine matches with circulating strains, low VE has recently been reported to be related to mutations in the egg-adapted H3N2 vaccine strain rather than to antigenic drift in circulating viruses [8,9].

Low VE against influenza A/H3N2 was reported during the 2012‒13 season, especially in the elderly (-11% and 9%) [10,11], even though the vaccine and epidemic strains matched. By contrast, during the 2013‒2014 season, influenza A(H1N1)pdm09 virus caused major epidemics in the United States (U.S.) and Canada, and high VE (60% to 75%) against influenza A(H1N1)pdm09 virus was reported [12,13]. The vaccine and epidemic strains also matched in the 2013‒2014 season. However, since there was a marked change in VE between the 2012‒2013 season and 2013‒2014 season because of the difference in epidemic influenza subtypes, it has become very important to estimate VE every season to monitor the performance of current influenza vaccine.

Although in many countries influenza vaccination is recommended starting at 6 months of age, there are few studies evaluating the effectiveness of trivalent inactivated influenza vaccine (TIV) in young children. TIV has been reported not to reduce the influenza A infection attack rate in 6–24 month-old children [14]. By contrast, a recent study revealed TIV was highly effective in children aged <24 months [15].

Indirect protection of the elderly by influenza vaccination of children and adults has recently been highlighted [16–19], and the indirect protection of the elderly is now thought to be more effective than direct protection. Assessing VE in children, especially in schoolchildren, is essential to achieving indirect protection of the elderly, because schools are the most efficient amplifiers of influenza epidemics in the community. The U.S. and Canada are aiming for universal immunization ranging from children 6 months of age to the elderly [20].

Influenza A(H1N1)pdm09 was the main strain in the 2013‒2014 season in Japan for the first time in 3 seasons [21]. Other epidemic viruses were influenza B and subtype A(H3N2). The number of patients/week/sentinel (epidemic index) exceeded 1.0 at the national level (a sign of the start of the epidemic season) in week 51 of 2013, and it was maintained at that level for 21 weeks till week 19 of 2014. The epidemic peaked in week 5 of 2014, when the incidence was 34.4 cases/sentinel.

In the 2013‒2014 season the prefectural and municipal public health institutes reported a total 8230 isolation/detections. The influenza viruses isolated/detected in the 2013‒2014 season consisted of A(H1N1)pdm09 (43%), subtype A(H3N2) (21%), and type B (36%). A(H1N1)pdm09 became dominant for the first time since the 2010‒2011 season [21]. The ratio of Yamagata lineage type B viruses to Victoria lineage type B viruses was 7:3.

In recent years, estimations of the effectiveness of influenza vaccine by a test-negative case-control design in Australia, Canada, New Zealand, the U.S., and European countries have been reported every year [8,9,11,12,15,22–27], and this design has become the standard design for assessing VE.

It is easy to perform a very large VE study in Japan, because all children with influenza-like illness who are seen in hospitals and clinics are tested by an influenza rapid detection test (IRDT) [28]. In this study we used the results of the IRDT as a basis for estimating VE by a test-negative control design in children who had received TIV during the 2013‒2014 season.

## Methods

### Study enrollment and location

Children (6 months to 15 years of age) with a fever of 38°C or over who received an IRDT in an outpatient clinic of one of 22 hospitals in Japan (in Gunma, Tochigi, Saitama, Tokyo, Chiba, Kanagawa, and Shizuoka prefectures) between November 9, 2013 and March 31, 2014 were enrolled in this study.

### Diagnosis of influenza

Nasopharyngeal swabs were obtained from all of the enrollees. Several IRDT different kits were used in the hospitals, including the Espline Influenza A&B-N kit (Fujirebio Inc., Tokyo, Japan), ImmunoAce FLU kit with LineJudge pdm kit (Tauns Laboratories, INC, Shizuoka, Japan), Quick Chaser Flu A, B kit (Mizuho Medy Co., Ltd., Saga, Japan), QuickNavi-Flu kit (DENKA SEIKEN Co., Ltd., Tokyo, Japan), and Clearline Influenza A/B/(H1N1)2009 kit (Alere Medical Co., Ltd., Tokyo, Japan). All of these IRDT kits are capable of differentiating between influenza A and influenza B. Four of the 22 hospitals used the Clearline Influenza A/B/(H1N1)2009 kit, or LineJudge pdm kit, which enables differentiate between influenza A, influenza B, and influenza A H1N1pdm09. All of the IRDT kits have similar sensitivities (88%‒100%) and specificities (94%‒100%) according to their manuals [29].

### Case and control patient identification

All participants who visited any of 22 hospitals located in the Kanto region in Japan between November 6 2013 and March 2014 were included in the study. Case patients were identified as patients who were IRDT-positive, and control patients were identified as patients who were IRDT-negative during the same period. Both the case patients’ and control patients’ medical charts were reviewed, and information regarding symptoms, influenza vaccination, number of vaccine doses (one or two), influenza complications and hospitalizations, gender, age, comorbidities, and treatment with neuraminidase inhibitors (NAIs) was collected and recorded. We excluded children for whom definite information on influenza vaccination was unavailable.

### Vaccine

A trivalent inactivated subunit-antigen vaccine is used in Japan. The vaccine strains to produce the vaccine for use in the 2013‒2014 season were A/California/7/2009(X-179A) for A(H1N1)pdm09, A/Texas/50/2012(X-223) for H3N2, and B/Massachusetts/02/2012(BX-51B) for type B, Yamagata lineage.

In Japan, two 0.25 ml doses of vaccine 2 to 4 weeks apart are recommended for children 6 months to 2 years of age, and two 0.50 ml doses of vaccine 2 to 4 weeks apart are recommended for children 3‒12 years of age. Only one 0.5 ml dose of vaccine is recommended for children over 13 years of age and over. The children in the vaccinated group in this study were immunized at our hospitals or elsewhere.

### Test-negative case-control design

VE was estimated by a test-negative case-control design in which a patient who presented with a fever of 38°C or over and was IRDT-positive for influenza virus was considered a case, and a patient who presented with a fever of 38°C or over and was IRDT-negative was considered a control. VE was defined as “1- OR (odds ratio)”, and OR was calculated as

(no. influenza-positive among vaccinated patients x influenza-negative among unvaccinated)

__________________________________

(no. influenza-negative among vaccinated patients x no. influenza-positive among unvaccinated patients).

We calculated VE and adjusted VE as shown in Statistical Analysis.

### Statistical Analysis

The statistical analysis was performed by using the SPSS. Ver. 22 software program (IBM, USA) and “Excel Tokei (Statistics) 2012 for Windows” software program (Social Survey Research Information Co., Ltd., Tokyo, Japan).

VE was adjusted for age group (6–11 months, 1–2 years, 3–5 years, 6–12 years and 13–15 years), comorbidity (yes or no), area of the Kanto region of Japan, i.e., (north area: Gunma Prefecture and Tochigi Prefecture; middle area: Saitama Prefecture, Tokyo Prefecture, and Chiba Prefecture; and south area: Kanagawa Prefecture and Shizuoka Prefecture), and month of onset of illness.

We also estimated VE according to the number of doses of vaccine administered, the phase of the season, and area of the Kanto region where the hospitals were located, and we assessed VE in preventing hospitalization. The Breslow Day test was used to assess the homogeneity of the odds ratios in several 2 × 2 contingency tables.

### Ethics

This study was approved by The Keio University Ethics Committee in 2013 (No. 20130216) and the Institutional Review Board (IRB) at each hospital. Eligible patients and their guardians (usually parents) were informed about the study objects and methods verbally at the outpatient departments. We recorded the necessary information from patients and the guardians using a standardized questionnaire sheets, when we obtained the consent to be enrolled to this study. The requirement for obtaining written consent was waived by IRBs because testing patients with IRDT is a standard practice in Japan.

## Results

### Characteristics of the enrollees

A total of 4970 children were enrolled in this study, but 243 were subsequently excluded from the analysis for the following reasons: 117 were <6 months old or >15 years old; 73 had a fever <38°C; 22 were examined twice during one episode (the data for the first visit for each patient was deleted); 30 had an unclear influenza vaccination history; and since one was both influenza A and B positive, there was a possibility of being false-positive. Of the remaining 4727 patients who were eligible for the analysis in this study (Table 1). 876 had influenza A, 66 of whom were confirmed to have A(H1N1)pdm09 infection, the other 810 patients had influenza A subtype unknown; and 1405 patients had influenza B. Of the 4727 subjects of the analysis, 2446 were IRDT-negative.

**Table 1:** Characteristics of Enrollees in 2013/14 Influenza Season.

	Any Influenza (%)		Type A (%)	H1N1pdm09^a^ (%)^a^	Type B (%)	Influenza Negative (%)	Difference between Any Influenza and Influenza Negative
**Sex**	**female**	1082 (47)	404 (46)	26 (39)	678 (48)	1096 (45)	
	**male**	1199 (53)	472 (54)	40 (61)	727 (52)	1349 (55)	
	**total**	**2281**	**876**	**66**	**1405**	**2445**	**P = 0.07** ^b^
**Age**	**6-11m/o**	49 (2)	39 (4)	2 (3)	10 (1)	166 (7)	
	**1–2 y/o**	342 (15)	224 (26)	21 (32)	118 (8)	803 (33)	
	**3–5 y/o**	539 (24)	248 (28)	17 (26)	291 (21)	675 (28)	
	**6–12 y/o**	1169 (51)	331 (38)	23 (35)	838 (60)	694 (28)	
	**13–15 y/o**	182 (8)	34 (4)	3 (5)	148 (11)	108 (4)	
	**total**	**2281**	**876**	**66**	**1405**	**2446**	**P<0.001** ^c^
**Comorbidity**	**No**	1802 (82)	692 (83)	39 (64)	1110 (81)	1874 (82)	
	**Yes**	401 (18)	146 (17)	22 (36)	255 (19)	413 (18)	
	**total**	**2203**	**838**	**61**	**1365**	**2287**	**P = 0.90** ^b^
**Province**	**north**	264 (12)	125 (14)	36 (55)	139 (10)	261 (11)	
	**middle**	1226 (54)	496 (57)	30 (45)	730 (52)	1356 (55)	
	**south**	791 (35)	255 (29)	0	536 (38)	829 (34)	
	**total**	**2281**	**876**	**66**	**1405**	**2446**	**p = 0.43** ^d^
**Months of Onset**	**Nov 2013**	0 (0)	0 (0)	0 (0)	0 (0)	12 (0)	
	**Dec 2013**	66 (3)	25 (3)	1 (2)	41 (3)	272 (11)	
	**Jan 2014**	609 (27)	424 (48)	32 (48)	185 (13)	629 (26)	
	**Feb 2014**	1017 (45)	345 (39)	27 (41)	672 (48)	838 (34)	
	**Mar 2014**	589 (26)	82 (9)	6 (9)	507 (36)	695 (28)	
	**total**	**2281**	**876**	**66**	**1405**	**2446**	**p<0.001** ^e^
**Visit (hours after onset)**	**<12 h**	695(32)	287 (35)	23 (35)	408 (30)	757 (33)	
	**12–48 h**	1312 (60)	498 (60)	40 (61)	814 (59)	1266 (55)	
	**>48 h**	187 (9)	39 (5)	3 (5)	148 (11)	281 (12)	
	**total**	**2194**	**824**	**66**	**1370**	**2304**	**p = 0.398** ^f^
	**>12 h**	1499	537	43	962	1547	
**Received Vaccine in 2013/2014**	**No**	1401 (61)	661 (70)	49 (74)	790 (56)	1143 (47)	
	**Yes**	880 (39)	265 (30)	17 (26)	615 (44)	1303 (52)	
	**total**	**2281**	**876**	**66**	**1405**	**2446**	**p<0.001** ^b^
**Vaccine Dose in 2013/2014**	**none**	1401 (62)	611 (70)	49 (74)	790 (56)	1143 (47)	
	**once**	219 (10)	71 (8)	5 (8)	148 (11)	270 (11)	
	**twice**	653 (29)	192 (22)	12 (18)	461 (33)	1010 (42)	
	**total**	**2273**	**874**	**66**	**1399**	**2423**	**p<0.001** ^g^
**Treatment with NAIs** *	**No**	72 (5)	22 (4)	0	50 (5)	1485 (96)	
	**Any**	1527 (95)	604 (96)	35 (100)	923 (95)	60 (4)	
	**total**	**1599**	**626**	**35**	**973**	**1545**	**p<0.001** ^h^

a Only four hospitals used IRDTs that can detect H1N1pdm09.

b Chi-square test.

c Mann–Whitney *U*- test.

d Chi-square test, Cramer's V = 0.0189.

e Chi-square test, Cramer's V = 0.1830.

f Chi-square test, comparing the number of patients who visited <12 hours after the onset with the number who visited later.

g number who visited later.

h Chi-square test, Cramer's V = 0.9160.

* Neuraminidase Inhibitors.

Table 1 shows the characteristics of the enrollees. The comorbidities consisted of respiratory comorbidities (n = 422), including asthma, neurological comorbidities (n = 128), including epilepsy, cardiac comorbidities (n = 57), allergic comorbidities (n = 49), renal comorbidities (n = 30), endocrinological comorbidities (n = 28), immunological comorbidities (n = 4), and other comorbidities (n = 96).

### VE against influenza

Influenza vaccine was effective against influenza virus infection overall (Table 2). It was more effective against influenza A infection than against influenza B infection (62% vs. 32%; *p* < 0.001, Breslow-Day test) and very effective against A(H1N1)pdm09, against which its VE was as high as 77% (95% confidence interval [CI]: 59 to 87).

**Table 2:** Effectiveness of Influenza Vaccine for Children in 2013/14 Influenza Season (N = 4727).

		Any Influenza^e^		Type A^e^		A(H1N1)pdm09 ^f^		Type B ^e^	
		VE% (95% CI)		VE% (95% CI)		VE% (95% CI)		VE% (95% CI)	
**All Ages 6 mo-15 y/o**	**Crude**	45 (38–51)	(880/2281) [1303/2446]*	62 (55–68)	(265/876) [1303/2446]*	77 (59–87)	(17/66) [281/468]*	32 (22–40)	(615/1405) [1303/2446]*
	**Adjusted** ^a^ ^,^ ^b^	45 (39–52)		63 (56–69)		77 (59–87)		26 (14–36)	
	**Adjusted** ^a^ ^,^ ^b^ ^,^ ^c^	45 (38–52)		63 (56–69)		77 (59–88)		25 (13–35)	
	**Adjusted** ^a^ ^,^ ^b^ ^,^ ^d^	51 (43–58)		67(59–74)		85 (65–93)		33 (20–45)	
**Age 6–11 m/o**	**Crude**	24 (-77-68)	(8/49) [34/166]	29 (-82-73)	(6/39) [34/166]	NA ^g^	(0/2) [8/36]	NA ^g^	(2/10) [34/166]
	**Adjusted** ^a^	21 (-87-67)		30 (-85-74)		NA ^g^		NA ^g^	
**Age 1–2 y/o**	**Crude**	61 (49–70)	(114/342) [451/803]	70 (58–78)	(63/224) [451/803]	67 (15–87)	(8/21) [102/157]	41(12–60)	(51/118) [451/803]
	**Adjusted** ^a^	63 (51–72)		72 (60–80)		67 (15–87)		41 (10–61)	
**Age 6 m/o-2 y/o**	**Crude**	55 (42–65)	(122/391) [485/969]	65 (52–74)	(69/263) [485/969]	60 (1–84)	(8/23) [110/193]	30 (-2-52)	(53/128) [485/969]
	**Adjusted** ^a^	57 (44–67)		67 (54–76)		62 (4–85)		29 (-6-52)	
**Age 3–5 y/o**	**Crude**	58 (48–67)	(208/539) [406/675]	72 (62–80)	(73/248) [406/675]	85 (44–96)	(3/17) [78/134]	43 (24–57)	(135/291) [406/675]
	**Adjusted** ^a^	60 (49–69)		73 (63–81)		84 (43–96)		44 (25–58)	
**Age 6–12 y/o**	**Crude**	36 (23–47)	(495/1169) [371/694]	55 (41–66)	(113/331) [371/694]	88 (64–96)	(5/23) [85/123]	27 (11–40)	(382/838) [371/694]
	**Adjusted** ^a^	39 (26–50)		58 (44–69)		90 (67–97)		30 (13–43)	
**Age 13–15 y/o**	**Crude**	29 (-17-57)	(55/182) [41/108]	32 (-57-70)	(10/34) [41/108]	NA ^g^	(1/3) [8/18]	29 (-21-58)	(45/148) [41/108]
	**Adjusted** ^a^	22 (-33-54)		12 (-115-64)		NA ^g^		23 (-34-56)	

a Adjusted for comorbidity (yes or no, except H1N1 analysis), area (north area, middle area, south of the Kanto region), months of onset.

b Adjusted for age (0–15 y/o).

c Adjusted for time tested after the onset (<12, 12–48 and >48 hours).

d Patients tested only >12 hours after onset.

Data for 3046 patients were available; 1499 for any influenza (A; 537, H1N1; 43, B; 962) and 1547 for influenza negative.

e Two hospitals have no information on comorbidity.

f Only four institutes used IRDTs that can detect A(H1N1)pdm09. One hospital had no information on comorbidity.

g Not analyzed because few patients developed influenza.

* (vaccinated/cases) [vaccinated/controls].

No statistically significant VE was detected in the infant group 6 months to 11 months of age, but the influenza vaccine was significantly more effective in the 1‒12-year-old group. Adjusted VE in the 1‒2-year-old group (63%, 95% CI: 51 to 72) and 3‒5-year-old group (60%, 95% CI: 49 to 69) was similar. VE was 36% (95% CI: 23 to 47) in the 6‒12-year-old group and significantly lower than the 58% (95% CI: 48 to 67) in the 3‒5-year-old group (*p* = 0.0049, Breslow-Day test).

By contrast, adjusted VE against influenza A(H1N1)pdm09 increased with the age group: from 67% (95% CI: 15 to 87) in the 1‒2-year-old group, to 84% (95% CI: 43 to 96) in the 3‒5-year-old group, and 90% (95% CI: 67 to 97) in the 6‒12-year-old group. No statistically significant VE was shown against influenza A, A(H1N1)pdm09, or influenza B in the 13‒15-year-old group.

Adjusted VE in the 6 months to 23 months group was 56% (95% CI: 38–69), 62% (95% CI: 43–75) against influenza A, 60% (95% CI: -35-88) against A(H1N1)pdm09, and 38% (95% CI: -9-64) against influenza B.

### Number of doses of vaccine

Two doses of influenza vaccine provided better protection against influenza A infection than only one dose in the group of children 6 months to 12 years of age (Table 3). The OR of two doses versus one dose was 0.72 (95% CI: 0.52 to 0.99). No significant difference in protection between the two doses was found against influenza B infection.

**Table 3:** Effectiveness of Influenza Vaccine for Children, by Vaccine Doses (6m/o-12y/o).

	Vaccine Doses	Cases	Controls	OR	95% CI
**Any Influenza**	**none**	1274	1076	0.62 (non vs once)	0.50–0.76
	**once**	172	235	0.54 (non vs twice)	0.48–0.62
	**twice**	646	1004	0.88 (once vs twice)	0.71–1.10
**Type A**	**none**	587	1076	0.48 (non vs once)	0.36–0.65
	**once**	62	235	0.35 (non vs twice)	0.29–0.42
	**twice**	191	1004	0.72 (once vs twice)	0.52–0.99
**A(H1N1)pdm** ^**a**^	**none**	47	177	0.38 (non vs once)	0.38–1.11
	**once**	4	40	0.20 (non vs twice)	0.10–0.38
	**twice**	12	228	0.53 (once vs twice)	0.16–1.71
**Type B**	**none**	687	1076	0.73 (non vs once)	0.57–0.94
	**once**	110	235	0.71 (non vs twice)	0.61–0.82
	**twice**	455	1004	0.97 (once vs twice)	0.75–1.25

a Among the 4 hospitals which used IRDTs that can detect H1N1pdm09.

### Protection against hospitalization

Influenza vaccination was effective in preventing hospitalization (Table 4), especially for influenza A virus infection (76%, 95% CI: 51 to 88). VE was as high as 90% (95% CI: 54 to 98) in preventing hospitalization for influenza A(H1N1)pdm09 infection. By contrast, the TIV was not effective in preventing hospitalization for influenza B virus infection.

**Table 4:** Effectiveness of Influenza Vaccine for Preventing Influenza Hospitalization.

	Immunization Status	No Hospitalization	Hospitalization with Influenza	Effectivenes for Preventing Influenza Hospitalization	95% CI
**Any Influenza**	**unvaccinated**	2080	65	51	24–69
	**vaccinated**	1778	27		
**Type A**	**unvaccinated**	2080	44	76	51–88
	**vaccinated**	1778	9		
**H1N1pdm09** ^**a**^	**unvaccinated**	223	15	90	54–98
	**vaccinated**	284	2		
**Type B**	**unvaccinated**	2080	21	0	-89-47
	**vaccinated**	1778	18		

a Among the 4 hospitals which used IRDTs that can detect A(H1N1)pdm09.

The reason for hospitalization was known in 14 (15%) of the 92 hospitalizations, and it was pneumonia in 5 cases, encephalopathy in 4 cases, asthma in 2 cases, mild consciousness disturbance in 1 case, seizure clusters in 1 case, and co-infection with *Mycoplasma pneumoniae* in 1 case.

### VE by month of onset of illness

VE was lower in the late phase of the influenza epidemic (Table 5), decreasing from 59% (95% CI: 49 to 66) in the 3-month period November, December, and January to 39% (95% CI: 29 to 47) in the 2-month period February and March. VE against influenza B was only 31% (95% CI: 19 to 40) in the 2-month period February and March. The number of influenza B patients was much higher in February and March, i.e., in the late phase of the epidemic.

**Table 5:** Effectiveness of Influenza Vaccine, by Phase.

	Any Influenza		Type A		A(H1N1)pdm09^a^		Type B	
	VE% (95%CI)		VE% (95% CI)		VE% (95% CI)		VE% (95% CI)	
**Nov, 2013-Jan, 2014**	59 (49–66)	(211/675) [478/913]*	66 (56–73)	(123/449) [478/913]*	78 (49–91)	(9/33) [82/130]*	42 (22–57)	(88/226) [478/913]*
**Feb-March, 2014**	39 (29–47)	(669/1606) [825/1533]	57 (46–66)	(142/427) [825/1533]	78 (49–90)	(8/33) [199/338]	31 (19–40)	(527/1179) [825/1533]
**Total**	45 (38–51)	(880/2281) [1303/2446]	62 (55–68)	(265/876) [1303/2446]	77 (59–87)	(17/66) [281/468]	32 (22–40)	(615/1405) [1303/2446]

a Among four hospitals which used IRDTs that can detect A(H1N1)pdm09.

* (vaccinated/cases) [vaccinated/controls].

### VE according to area and hospital

There were no significant differences between VE against influenza A according to area, but VE against influenza B was significantly higher in the north area than in the middle area or south area (55% in the north area vs. 27–28% (both p < 0.05) (Table 6). The VE values according to hospital ranged widely from -275% to 84% against influenza B. By contrast, VE values against influenza A were similar in most of the hospitals.

**Table 6:** Effectiveness of Influenza Vaccine in Children (6 m/o-12 y/o) According to Hospital (A to V).

		Any Influenza		Type A		A(H1N1)pdm09 ^a^		Type B		Influenza Negative		VE (95%CI)			
Area		vaccine^b^	no^c^	vaccine^b^	no^c^	vaccine^b^	no^c^	vaccine^b^	no^c^	vaccine^b^	no^c^	Any Influenza	Type A	A(H1N1)pdm09 ^a^	Type B^d^
**North**	**A**	41	68	15	36	7	29	26	32	116	77	60 (35–75)	72 (46–86)	84 (62–93)	46 (2–70)
	**B**	20	47	7	22			13	25	4	3	68 (-56-93)	76 (-33-96)		61 (-101-92)
	**C**	28	60	14	31			14	29	31	30	55 (11–77)	56 (2–80)		53 (-5-79)
**North Total**		**89**	**175**	**36**	**89**	**7**	**29**	**53**	**86**	**151**	**110**	**63 (47–74)**	**71 (53–81)**	**82 (62–93)**	**55 (32–71)**
**Middle**	**D**	20	23	4	13	0	4	16	10	58	54	19 (-64-60)	71 (7–91)	100	-49 (-257-38)
	**E**	49	105	14	63			35	42	99	92	57 (33–72)	79 (61–89)		23 (-32-54)
	**F**	14	33	4	17			10	16	37	36	59 (10–81)	77 (25–93)		39 (-52-76)
	**G**	99	152	46	71			53	81	126	90	53 (33–68)	54 (27–71)		53 (27–70)
	**H**	79	55	20	24	6	10	59	31	65	34	25 (-29-56)	56 (10–79)	69 (6–89)	0 (-82-45)
	**I**	23	20	12	11	4	6	11	9	42	22	40 (-33-73)	43 (-50-78)	65 (-37-91)	36 (-78-77)
	**J**	78	67	19	23			59	44	79	41	40 (0–63)	57 (12–79)		30 (-20-60)
	**K**	55	95	19	43			36	52	102	92	48 (19–66)	60 (27–78)		38 (-4-62)
	**L**	14	23	10	16			4	7	41	44	35 (-44-70)	33 (-65-73)		39 (-125-83)
	**M**	82	140	17	50			65	90	106	96	47 (22–64)	69 (43–83)		35 (0–57)
**Middle Total**		**513**	**713**	**165**	**331**	**10**	**20**	**348**	**382**	**755**	**601**	**43 (33–51)**	**60 (51–68)**	**67 (26–85)**	**27 (13–39)**
**South**	**N**	78	194	21	85			57	109	111	150	46 (22–62)	67 (43–80)		29 (-6-53)
	**O**	73	141	11	25			62	116	90	123	29 (-5-52)	40 (-29-72)		27 (-10-52)
	**P**	8	16	2	7			6	9	41	61	26 (-90-71)	57 (-115-92)		1 (-200-67)
	**Q**	7	7	2	5			5	2	6	9	-50 (-553-66)	40 (-317-91)		-275 (-2504-46)
	**R**	45	40	9	25			36	15	68	29	52 (12–74)	85 (63–94)		-2 (-115-51)
	**S**	42	74	10	24			32	50	36	34	46 (2–71)	61 (6–84)		40 (-15-68)
	**T**	2	6	0	1			2	5	10	4	87 (4–98)	100		84 (-19-98)
	**U**	14	23	7	14			7	9	17	15	46 (-40-79)	56 (-38-86)		31 (-130-79)
	**V**	9	12	2	5			7	7	18	7	71 (0–91)	84 (0–98)		61 (-52-90)
**South Total**		**278**	**513**	**64**	**191**	**0**	**0**	**214**	**322**	**397**	**432**	**41 (28–52)**	**64 (50–73)**		**28 (10–42)**
**Total**		**880**	**1401**	**265**	**611**	**17**	**49**	**615**	**790**	**1303**	**1143**	**45 (38–51)**	**62 (55–68)**	**77 (59–87)**	**32 (22–40)**

a Among four hospitals which used IRDTs that can detect A(H1N1)pdm09.

b Number of patients vaccinated.

c Number of patients unvaccinated.

d VE against any influenza and VE against influenza B were higher in the north area than in the middle or south area (Breslow-Day, p < 0.05).

### Vaccine coverage

The proportion of vaccine coverage calculated for the IRDT-negative enrollees was 53% (1303/2446). By age group, it was: 6‒11 months, 21% (34/166); 1‒5 years, 58% (857/1478); for 6‒12 years, 53% (371/694); and 13‒15 years, 38% (41/108).

## Discussion

In this large study of over 4700 children 6 months to 15 years of age the overall influenza VE for prevention of laboratory-confirmed medically attended influenza illness was 46% (Table 2). High VE was shown in the influenza A group (63%), and VE was as high as 77% in the group with confirmed A(H1N1)pdm09 infection. Our results were consistent with reports in other countries where A(H1N1)pdm09 was the main epidemic virus [12,13]. In the 2013‒2014 season in Japan, both A(H3N2) and A(H1N1)pdm09 were co-circulating, but A(H1N1)pdm09 was the dominant strain [21].

VE against influenza A was over 72% in the group of children 1‒5 years of age (72% in the 1‒2-year-old group, 73% in the 3‒5-year-old group) and was higher than VE in the 6‒12-year-old group (58%) (Table 2). By contrast, VE against A(H1N1)pdm09 increased with the age groups, from 67% in the 1‒2-year-old group, to 84% in the 3‒5-year-old group, and 90% in the 6‒12-year-old group. Most of the children in the 6‒15-year-old group had been infected in the A(H1N1)pdm09 pandemic in 2009‒2010 [30] and probably had sufficient residual immunity against A(H1N1)pdm09 from five years before. The pre-epidemic measurement of hemagglutination inhibition (HI) titers showed that 60%‒70% of the children 5‒15 years of age in Japan had HI titers to A(H1N1)pdm09 that were over 1:40 [31]. Because no antigenic changes in A(H1N1)pdm09 have been reported, the majority of children over 6 years of age who were IRDT-positive for influenza A probably had an A(H3N2) infection, and that would have led to the lower effectiveness against influenza A in this age group. In other studies VE has been estimated to be low in patients infected with A/H3N2 [8,9]. VE has not been demonstrated in children 13‒15 years of age, because the number of children tested was small and/or they had mainly contracted influenza A/H3N2.

VE against influenza B in the present study was low, only 26% (Table 2). Lower vaccine efficacy against influenza B than against influenza A has been postulated in Japan, especially in young children [4]. Low VE against influenza B probably reflects the characteristic epidemic pattern of influenza B in Japan: influenza B virus epidemics usually start in February, after the end of the influenza A epidemic, and continue through March to April. Since children in Japan usually receive influenza vaccine in October and November, the level of antibody titer generated in response to the vaccine may be lower in March when influenza B epidemics peak. Our data showed that VE against influenza A and B was lower in February and March (Table 5). The lower VE against influenza B was probably attributable to waning immunity in response to the vaccine, as has been reported in regard to the effectiveness of vaccines against influenza A H3N2 [26,32], and the low VE against influenza B was partly attributable to the mixed epidemic of influenza B lineages, i.e., of B/Victoria and B/Yamagata, in the 2013‒2014 season in Japan [21], although B/Yamagata was both the main epidemic strain and the vaccine strain. B-lineage mismatches present a greater obstacle to vaccine efficacy in children [3]. In the near future, quadrivalent vaccines that contain both B-lineage antigens will be introduced in Japan, thereby reducing B-lineage mismatches. Low HI titer responses to influenza B strain are of particular concern in children who receive influenza vaccine [3], because influenza B infection is common in children and as serious clinically as influenza A infection. NAIs are also known to be less effective against influenza B [33].

In this study influenza vaccine was not effective against either influenza A (30%, 95% CI -85, 74) or influenza B (-45%, 95% CI -684, 75) in 6- to 11-month-old infants, but it was effective in children over 1 year old (Table 2). Indirect protection attributable to the mass vaccination program of schoolchildren in Japan in the 1960s to 1980s protected young children 1–4 years of age against influenza encephalopathy [17,19]. Young children did not receive influenza vaccine in the 1960s to 1980s. On the other hand, traditional cohort studies to estimate VE may have yielded excessively high VE for infants. One study reported 42%-69% VE in infants [34]. Since siblings and/or parents in families in which an infant has received influenza vaccine have usually also received influenza vaccine, thereby providing highly effective indirect protection to their infants whether indirect protection exists in the background should be noted when interpreting reports on VE in children.

Two doses of influenza vaccine have been reported to be necessary to provide sufficient protection in children, especially in young children [6,7,35,36]. However, the results of the present study showed that only one dose of influenza vaccine was effective in protecting against influenza A (52%) and influenza B (27%). Two doses of influenza vaccine were needed to optimize protection only against influenza A in the present study (one dose vs. two doses, OR = 0.72) (Table 3). However, a second dose did not have any additive effect against influenza B.

A meta-analysis of VE in children showed no convincing evidence that influenza vaccine can reduce mortality, hospitalizations, or serious complications [37]. However, the results of the present study demonstrated that influenza vaccination was effective in reducing hospitalization of children infected with influenza A infection by as much as 76% and of children infected with A(H1N1)pdm09 infection by as much as 90% (Table 4). On the other hand, no effectiveness in preventing hospitalization for influenza B was shown. By contrast, in the 2002‒2008 seasons VE for preventing hospitalization with influenza A and B in children 6 months to 5 years old was reported to be 71% and 72% [34]. There is a recent report of a study showing that influenza vaccination was associated with a three-quarters reduction in the risk of life-threatening influenza illness in children 6 months to 17 years of age [38]. Moreover, VE against hospitalization with laboratory-confirmed influenza A and B has been estimated to be 61.7% [39].

The results of this study showed clear differences between VE against influenza B according to area (Table 6). In the 2013‒2014 season, the ratio of Yamagata lineage viruses to Victoria lineage viruses was 7:3 nationwide [21]. Since the vaccine virus was the Yamagata lineage in the 2013–2014 season, Yamagata lineage viruses may have been more epidemic in the north area than in the other areas of the Kanto region.

The limitations of this study need to be considered. Unlike most previous test-negative case-control design studies based on PCR data, this study was based on the results of IRDTs. Suzuki et al. [40] found no difference between VE estimated on the basis of IRDT results on the basis of PCR data. However, the use of IRDTs in test-negative studies may result in underestimations of VE. Orenstein et al. [41] reported that the bias toward underestimating true VE introduced by low test specificity increases and lesser degree, by low test sensitivity. In a case-control study on influenza VE based IRDT results [5], it was postulated that virus shedding is greatest during the first days of influenza infection and therefore, patients who were tested during the early phase of the illness were more likely to test positive. In our study, about 90% of the enrollees received IRDT within 48 hours of onset of illness (Table 1).

On the other hand, the sensitivity of IRDTs is low when patients are tested within 12 hours after the onset of influenza illness because of the low virus infection titers in the upper respiratory tract in the early phase of infection [42]. We therefore selected patients who were tested 12 hours or more after the onset of influenza illness, and when we estimated the VE of the influenza vaccine in that group of patients, we found that it was slightly higher (+4% to +8%) (Table 2). When estimating VE by a test-negative case-control design based on IRDT results, it is better to select patients tested 12 hours or more after the onset of influenza-like illness.

Almost all children with influenza-like illness in Japan receive an IRDT, and if positive, they are treated with NAIs [28]. This diagnosis and treatment system established in Japan worked well during the pandemic caused by A(H1N1)pdm09 and resulted in the very low fatality rate in Japan during that pandemic [30]. In future epidemics in Japan, VE estimated by a test-negative case-control design based on IRDT results from various parts of Japan will be reported rapidly. The large number of cases in Japan makes it possible to estimate VE with considerable precision, and the most appropriate vaccination policy should be established based on the data obtained.
